# Adding Walnuts to the Usual Diet Can Improve Diet Quality in the United States: Diet Modeling Study Based on NHANES 2015–2018

**DOI:** 10.3390/nu15020258

**Published:** 2023-01-04

**Authors:** Lisa A Spence, Beate Henschel, Rui Li, Carmen D Tekwe, Krisha Thiagarajah

**Affiliations:** 1 Department of Applied Health Science, School of Public Health-Bloomington, Indiana University, Bloomington, IN 47905, USA; 2 Department of Epidemiology and Biostatistics, School of Public Health-Bloomington, Indiana University, Bloomington, IN 47905, USA

**Keywords:** NHANES, walnuts, modeling, diet quality, Healthy Eating Index

## Abstract

Background: The under-consumption of calcium, potassium, fiber, and vitamin D is considered a U.S. public health concern. Shifts in eating patterns that increase the consumption of vegetables, fruits, whole grains, nuts/seeds, and dairy products can help achieve the recommended intakes of these nutrients, leading to healthier diets. Objective: We assessed the impact of adding 1 ounce (28.35 g) of walnuts to usual diets on diet quality and nutrients of concern, including magnesium, fiber, and potassium. Methods: We utilized 24 h dietary recalls obtained from the What We Eat in America, National Health and Nutrition Examination Survey (NHANES) and modeled the addition of 1 ounce (28.35 g) of walnuts to the usual diets of no-nut consumers. No-nut consumers aged ≥4 years (*n* = 7757) from the 2015–2018 NHANES study were included. Population percentages with intakes below the estimated average requirement (EAR) values for calcium, magnesium, folate, and vitamin E and above the adequate intake (AI) values for potassium and fiber were examined. Diet quality was assessed using the Healthy Eating Index-2015 (HEI-2015). The National Cancer Institute method was used to estimate the usual and modeled intakes. Significant differences between usual (current) and modeled intakes were determined using non-overlapping 95% confidence intervals. All analyses included sample weights to account for the NHANES survey design. Results: Adding 1 ounce (28.35 g) of walnuts to the usual diet resulted in significant reductions in the percentages of adults with intakes below the EAR for magnesium and folate (69.6% vs. 52.0%; 49.2% vs. 40.6%, respectively), and increased the percentage of adults above the AI for potassium (22.8% vs. 26.5%). A similar trend was observed among children (4–18 years). HEI scores improved significantly from 49.1 (95% CI: 48.0–50.4) to 58.5 (95% CI: 57.5–59.6) in children and from 52.4 (95% CI: 51.0–53.8) to 59.2 (95% CI: 58.0–60.5) in adults. Conclusions: Adding 1 ounce (28.35 g) of walnuts to the usual diet of no-nut consumers improved the diet quality and adequacy of some under-consumed nutrients.

## 1. Introduction

The 2020–2025 Dietary Guidelines for Americans [1] report that the inadequate intake of nutrient-dense foods and beverages across food groups in modern diets has resulted in the under-consumption of some nutrients and dietary components. The under-consumption of calcium, potassium, dietary fiber, and vitamin D is considered a public health concern for the U.S. population because the inadequate intake of these dietary components is associated with major health concerns such as hypertension, cardiovascular disease, and certain types of cancer [1]. Shifts in eating patterns that increase the consumption of vegetables, fruits, whole grains, nuts or seeds, and dairy products can move consumers closer to achieving the recommended intakes of these nutrients of concern [1], which can lead to a healthier diet.

Walnuts are rich in fiber, potassium, calcium, magnesium, folate, vitamin E, phytosterols, polyphenols, and polyunsaturated fatty acids (PUFAs) [2]. Walnuts are especially rich in alpha-linolenic acid (ALA) and linoleic acid [3]. A 1-ounce serving (28.35 g) of walnuts provides 18 g of total fat, 2.5 g of monounsaturated fat, 13 g of PUFA (including 2.5 g of ALA, a plant-based anti-inflammatory omega-3 PUFA), 2 g of fiber, and 4 g of plant protein [4]. The unique nutrient composition of walnuts differs from those of other nuts because approximately 10% of the energy provided by walnuts comes in the form of ALA, and walnuts are a rich source of phytomelatonin and possess more polyphenols than other types of nuts [5]. The consumption of walnuts may improve overall health because the unique bioactive compounds found in walnuts likely impact several metabolic pathways [6]. The fatty acid composition of walnuts may provide cardioprotective effects by improving lipids’ profile, in addition to antithrombotic, anti-inflammatory, and vasculoprotective effects, resulting in improved endothelial function and reduced atherosclerosis [7,8,9]. Including walnuts as a source of plant protein in the diet could also increase the intake of several under-consumed nutrients, in addition to increasing the intake of omega-3 PUFA, thus potentially contributing to improved cardiovascular health. The polyphenols found in walnuts may also play a role in reducing low-density lipoprotein (LDL)-cholesterol, adiposity, and blood glucose levels, which has promising implications for reducing the impacts of cardiovascular disease and diabetes [10]. Nutrients such as vitamin E, potassium, magnesium, and calcium found in walnuts have been associated with healthier levels of inflammation, blood pressure, and insulin sensitivity [11].

The current study modeled the effects of adding walnuts to the typical U.S. diet in a population of no-nut consumers. Investigators hypothesized that the incorporation of walnuts in these models would result in improvements in nutritional adequacy and diet quality, including increases in the intake of fiber, potassium, calcium, magnesium, vitamin E, and omega-3 PUFA. Investigators also hypothesized that the addition of walnuts to the daily diet would result in larger percentages within subpopulations stratified by age and sex that would meet the recommended nutrient and dietary intake levels established by the Food and Nutrition Board of the Institute of Medicine, National Academy of Sciences and U.S. Dietary Guidelines for Americans.

## 2. Materials and Methods

### 2.1. Study Population 

**Figure 1:**
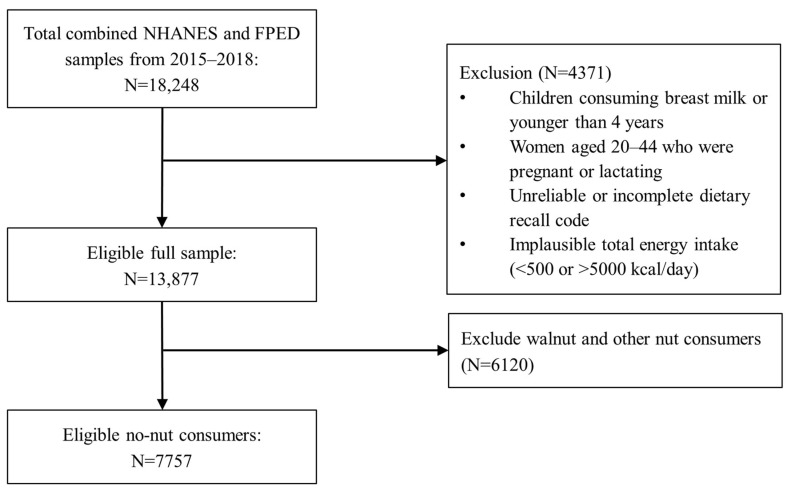
Flow chart of the sample selection from the NHANES 2015–2018.

The National Health and Nutrition Examination Survey (NHANES) is a nationally representative, cross-sectional survey of U.S. non-institutionalized civilian residents. NHANES data are collected by the National Center for Health Statistics of the Centers for Disease Control and Prevention. Written informed consent was obtained for all participants or their proxies, and the survey protocol was approved by the Research Ethics Review Board at the National Center for Health Statistics. Dietary data were obtained from two NHANES cycles: 2015–2016 and 2017–2018. Children consuming breast milk or younger than 4 years, women aged 20–44 years who were pregnant or lactating, respondents with unreliable food recall data, and individuals reporting implausibly low (<500 kcal/d) or high (>5000 kcal/d) caloric intakes were excluded from the study [12]. Previous studies indicated that tree nut consumption increased in recent times and was associated with improved diet quality with a wide range of health benefits [13,14]. Given this knowledge of improved diet quality among nut consumers, we are focusing on no-nut consumers in this study; therefore, nut consumers were excluded in the modeling analyses. The sample of no-nut consumers included 2670 children and adolescents (age 4–18 years) and 5087 adults (19 years and older), as summarized in Figure 1.

### 2.2. Dietary Intake Measures

Data from two non-consecutive 24 h dietary recalls were used. The first 24 h dietary recall was completed in-person at the Mobile Examination Center (MEC) with a trained interviewer [15]. The second dietary recall was completed over the phone 3–10 days later. Parents or guardians provided 24 h dietary recalls for children aged 2–5 years; children aged 6–11 years were assisted by an adult; all others provided their own dietary recalls. Only dietary recall data considered complete and reliable by the United States Department of Agriculture (USDA) Food Surveys Research Group were included in the present study. Detailed descriptions of the applied dietary interview methods can be found in the NHANES Dietary Interviews Procedure Manual [15]. In our sample of no-nut consumers, 79% of respondents completed two dietary recalls.

### 2.3. Food Composition Data

Nutrient data were obtained from the Food and Nutrient Database for Dietary Studies (FNDDS) 2015–2016 [16] for 2015–2016 NHANES data and from FNDDS 2017–2018 for 2017–2018 NHANES data [17]. Data on the consumption of food groups of interest, including protein foods, were obtained from the Food Patterns Equivalent Database (FPED). Each NHANES data cycle was analyzed using the corresponding cycle-specific version of FPED (for example, FPED 2017–2018 was used for 2017–2018 NHANES data) [18,19].

### 2.4. Walnut Consumption Classification 

Based on food items that NHANES respondents consumed and the FNDDS ingredient list, walnut consumers were identified when respondents consumed food items containing walnuts (ingredient codes 12155 “Nuts, walnuts, English” and 12154 “Nuts, walnuts, black, dried”) [17]. Other nut consumers were identified using the FPED [18,19] when respondents reported non-zero amounts in the component “Nuts and Seeds (Peanuts, tree nuts, and seeds (ounce equivalent); excludes coconut)” and were not already counted as a walnut consumer. All remaining respondents were considered no-nut consumers. We present demographic characteristics for the three different nut consumption groups (Table 1), but only no-nut consumers were included in the modeling study.

### 2.5. Modeling

Investigators modeled the nutritional impact of adding 1 ounce (28.35 g) of walnuts to participants’ diets. The primary outcome measures were changes in the intake of nutrients of public health concern identified by the 2020–2025 U.S. Dietary Guidelines for Americans, including potassium, dietary fiber, and magnesium. Additionally, the Dietary Guidelines for Americans recommends limiting saturated fat intake and increasing the consumption of unsaturated fatty acids. Investigators hypothesized that walnuts could supplement nutrients being consumed below the recommended intake values such as fiber, potassium, and magnesium and could increase intakes of polyunsaturated fatty acids. According to the USDA, 1 ounce (28.35 g) of walnuts contains 185 kcal, 4.32 g of protein, 1.74 g of saturated fat, 2.57 g of omega-3 PUFA (ALA), 10.8 g of omega-6 PUFA (Linoleic acid), 27.8 mg of calcium, 44.8 mg of magnesium, 27.8 µg dietary folate equivalents (DFE), 0.198 mg of vitamin E, 125 mg of potassium, 0.451 mg of copper, 0.876 mg of zinc, and 1.9 g of fiber [20].

The 2015 Healthy Eating Index (HEI-2015) was examined as a composite measure of diet quality. The HEI-2015 is an energy-adjusted measure of diet quality based on 13 components, including 9 components that are encouraged (total fruit, whole fruit, total vegetables, greens and beans, whole grains, dairy, total protein, protein from plant and seafood sources, and the fatty acid ratio (favoring a higher ratio of monounsaturated fatty acids and PUFAs to saturated fatty acids)) and four components that are discouraged (refined grains, energy from saturated fat, added sugars, and added sodium). HEI scores range from 0 to 100; higher scores indicate better dietary alignment with Dietary Guidelines for Americans recommendations. Details on the HEI-2015 algorithm have been described previously [21]. A version of the HEI corresponding to the 2020–2025 Dietary Guidelines for Americans is not yet available, but the 2020–2025 Dietary Guidelines for Americans and 2015–2020 Dietary Guidelines for Americans are generally comparable regarding which nutrients and food groups should be encouraged or limited [1,22]. Therefore, we used the HEI-2015 to assess diet quality.

### 2.6. Analyses

The National Cancer Institute (NCI) method [23,24] was used to estimate the usual dietary intake of selected nutrients, and this estimation was repeated after the addition of 1 ounce (28.35 g) of walnuts to the usual dietary intake for a specific gender and age group. The NCI method requires the use of balanced repeated replication weights to account for the complex survey design used by the NHANES. The NCI macros Mixtran (version 2.1), Distrib (version 2.1), and brr_pvalue_ci_macro (version 1.0) were used to obtain the usual intake estimates [25]. Covariates in the NCI method include the sequence of a participant’s intake (Day 1 or Day 2), age, gender, race, energy intake, and a variable for weekday/weekend consumption (Monday–Thursday: weekday, Friday–Sunday: weekend). For each nutrient of interest, the population means and corresponding 95% confidence intervals (CIs) were calculated. The percentages of the population with intakes below the estimated adequate requirement (EAR) for protein, calcium, magnesium, copper, zinc, folate, and vitamin E were determined. Additionally, the percentages of the population with intakes above the recommended adequate intake (AI) for potassium, fiber, and ALA were examined, as recommended by the published Dietary Reference Intakes [26]. 

The HEI-2015 score was estimated using the population ratio method described previously [27], using data from the first 24 h recall (day 1). Investigators used the SAS macro provided by the NCI (hei2015.score.macro.sas) [27] and adapted it for the 2015–2016 and 2017–2018 NHANES and FPED cycles used in this study. The modeled HEI score was based on the reported intake plus the nutrient contents found in 1 ounce (28.35 g) of walnuts [21] for the following components: monounsaturated fatty acids (2.53 g), PUFAs (13.4 g), saturated fats (1.74 g), energy intake (185 calories), total protein (2-ounce equivalent), seafood and plant proteins (2-ounce equivalent), and sodium (0.567 mg). 

The results for the usual (current) and modeled nutrient intake and HEI scores were presented by age groups (4–8 years, 9–13 years, 14–18 years, 19–50 years, 51–70 years, 71 years and older) and by gender. Significant differences between the usual (current) and modeled intakes (nutrients, percent below EAR or above AI, HEI scores) were determined using non-overlapping 95% CI. We used SAS software (version 9.4, SAS Institute Inc., Cary, NC, USA) for all data analyses.

## 3. Results

### 3.1. Demographics and Walnut Consumption

Table 1 displays the weighted population demographics and characteristics of the combined population for the 2015–2016 and 2017–2018 combined NHANES cycles (*n* = 13,877), which include a representative sample of the U.S. population, according to the consumption of walnuts, other nuts, or no-nuts. Only a small percentage of the U.S. population regularly consumed walnuts (7%). Among this small subpopulation, females and older individuals consumed more walnuts than men and younger adults. Additionally, higher percentages of white individuals (11.0%), those with higher annual incomes (>$100,000, 12.3%), and those with more education (college degree or above, 13.4%) consumed walnuts than other groups. The family income to poverty threshold ratio was highest (mean ± SE: 3.52 ± 0.10) for walnut consumers compared to other nut consumers (3.23 ± 0.06) and no-nut consumers (2.52 ± 0.06).

### 3.2. Impact of Walnut Consumption on Nutrient Adequacy

Gaps between the actual and recommended consumption levels in the typical U.S. diet are currently reported for calcium, magnesium, vitamin E, potassium, and fiber; therefore, the percentages of individuals consuming above the AI for potassium and fiber and below the EAR for all other nutrients of concern were examined for both the usual (current) and modeled intakes (i.e., with the addition of 1 ounce of walnuts), and the results are presented in Table 2, Table 3 and Table 4. Table 2 shows the usual (current) and modeled mean intake values for seven under-consumed nutrients, magnesium, calcium, potassium, folate, vitamin E, fiber, and omega-3 fatty acids, in addition to overall protein and caloric intakes. Table 3 and Table 4 show the results for selected nutrients expressed as the percentage of the U.S. population consuming either below the EAR (calcium, magnesium, folate, vitamin E; Table 3) or above the AI (potassium and fiber; Table 4), stratified by age and sex.

#### 3.2.1. Magnesium 

Among children and adolescents (aged 4–18 years), the mean intake of magnesium was 219.2 mg (95% CI: 214.2 mg–224.2 mg). Unsurprisingly, adding 1 ounce (28.35 g) of walnuts, which contained 44.8 mg of magnesium, to the diet significantly improved the intake of magnesium (263.9 mg, 95% CI: 259.0 mg–268.9 mg, Table 2). A similar significant increase in magnesium intake was observed among adults in all three age groups. The prevalence of inadequate magnesium intake was highest among adolescents aged 14–18 years (82.7% of boys and 89.0% of girls, Table 3). Although adding 1 ounce (28.35 g) of walnuts to the diet significantly improved the magnesium intake among all age categories, the prevalence of inadequate magnesium intake remained high among adolescent boys and girls (14–18 years), men aged 51–70 years, and adults aged >71 years. The prevalence of inadequate magnesium intake was higher among adult men than adult women, whereas in children, the prevalence of inadequate magnesium intake was higher among girls than boys.

#### 3.2.2. Folate

The modeled mean intake of folate, after the addition of 1 ounce (28.35 g) of walnuts (containing 27.8 µg DFE of folate) to the diet, significantly improved from 347.5 µg DFE (95% CI: 338.2 µg DFE–356.8 µg DFE) to 375.3 µg DFE (95% CI: 366.0 µg DFE–384.5 µg DFE) among children and adolescents. A similar trend was observed among adults aged 19–50 years and 51–70 years (Table 2). Girls had a higher prevalence of inadequate folate intake than boys within each age group (5.6%, 24.5%, and 63.9% among girls and 4.0%, 19.1%, and 44.6% among boys for ages 4–8, 9–13, and 14–18 years, respectively). In our model, adding 1 ounce (28.35 g) of walnuts to the diet significantly reduced folate inadequacy among individuals aged 4–18 years (28.3% to 22.0%, Table 3). At the usual (current) intake, adult women (age > 19 years) had a higher prevalence of inadequate folate intake (61.6%) than adult men (age > 19 years, 38.2%). For women aged 19–50 years, the prevalence of inadequate folate intake was 58.4%, but the addition of 1 ounce (28.35 g) of walnuts to the diet significantly reduced folate inadequacy to 48.1%. The addition of 1 ounce (28.35 g) of walnuts to the diet significantly reduced the prevalence of inadequate folate intake across all age and sex categories for adults, except men aged 19–50 years and adults aged >71 years.

#### 3.2.3. Potassium

Adding 1 ounce (28.35 g) of walnuts that contained 125 mg of potassium to the usual diet of no-nut consumers significantly improved the mean potassium intake for children and adolescents aged 4–18 years, from 2070 mg (95% CI: 2019 mg–2121 mg) to 2195 mg (95% CI: 2144 mg–2246 mg), as shown in Table 2. Our estimates indicate that approximately 17.3–31.7% of Americans currently consume above the AI for potassium, satisfying the recommended potassium intake (Table 4). Adding 1 ounce (28.35 g) of walnuts to the usual diet significantly increased the proportion of children aged 4–18 years with potassium intakes above AI, from 25.4% to 30.4%, and increased the proportion of adults (aged >19 years) with potassium intakes above AI from 22.8% to 26.5%. Overall, adding 1 ounce (28.35 g) of walnuts to the usual diet improved potassium intake; however, the total proportion of individuals with potassium intakes above AI remained at 26.5% for adults aged >19 years and at 30.4% for children aged 4–18 years, indicating that much of the U.S. population would continue to have an intake less than the recommended potassium amount.

#### 3.2.4. Fiber

In general, adding 1 ounce (28.35 g) of walnuts to the usual diet significantly increased the mean intake of fiber for every age and sex group (Table 2). Fewer than 2.4% of Americans consume above the AI for fiber. 

#### 3.2.5. Other Vitamins, Minerals, and Omega-3 Fatty Acids

The impacts of adding 1 ounce (28.35 g) of walnuts to the diet were also modeled for the intake of zinc, copper, selenium, and omega-3 fatty acids. For copper, the prevalence of inadequate intake ranged from 0.2% (boys aged 4–8 years) to 29.4% (girls aged 14–18 years). The prevalence of inadequate zinc intake varied across age and sex groups (boys aged 4–8 years: 0.4%; adolescent girls aged 14–18 years: 32.3%; women aged 19–50 years: 19.7% for females; adults > 71 years: 34.5%). The model that included the addition of 1 ounce (28.35 g) of walnuts to the diet indicated significant reductions in the prevalence of inadequate zinc intake among most age and sex groups, except for boys aged 4–8 and 9–13 years. The model demonstrated that adding 1 ounce (28.35 g) of walnuts to the diet completely resolved the copper inadequacy for most age and sex groups. 

The prevalence of inadequate vitamin E intake was high among most age and sex categories with the exception of young children aged 4–8 years (Table 3). Adding 1 ounce (28.35 g) of walnuts, which contains 0.198 mg of vitamin E, to the diet resulted in only a small, not statistically significant reduction in the prevalence of inadequacy among all age and sex groups, leaving the prevalence of inadequate vitamin E intake essentially unchanged at 80.7–94.8% among adults.

### 3.3. Diet Quality

**Figure 2:**
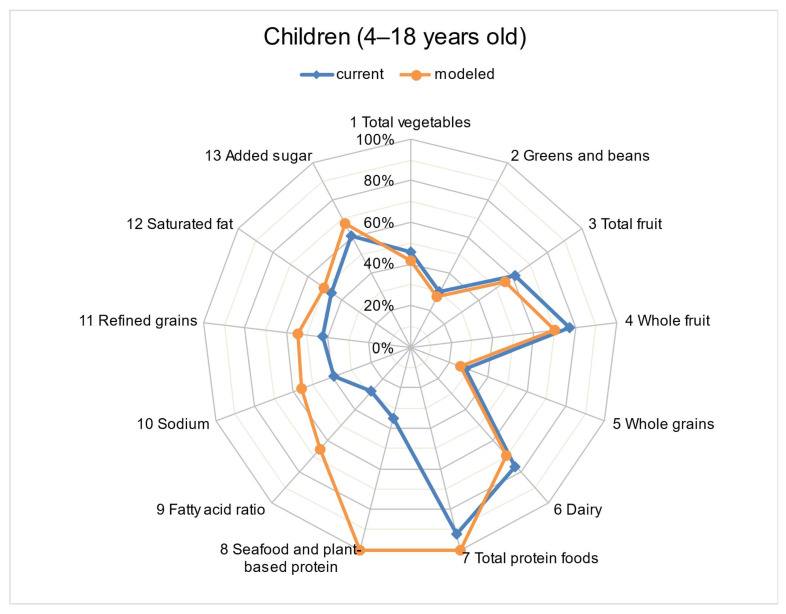
Radar plot to compare HEI-2015 * component scores (% of maximum possible score) between current intake and modeled intake (addition of 1 ounce of walnuts) among children aged 4–18 years. * HEI-2015 includes 13 dietary components. Nine adequacy components (those recommended for inclusion in a healthy diet) include total vegetables (5), greens and beans (5), total fruits (5), whole fruits (5), whole grains (10), dairy (10), total protein foods (5), seafood and plant proteins (5), and fatty acids ratio (10). Four moderation components (those that should be consumed sparingly) include sodium (10), refined grains (10), saturated fats (10), and added sugars (10) (21).

**Figure 3:**
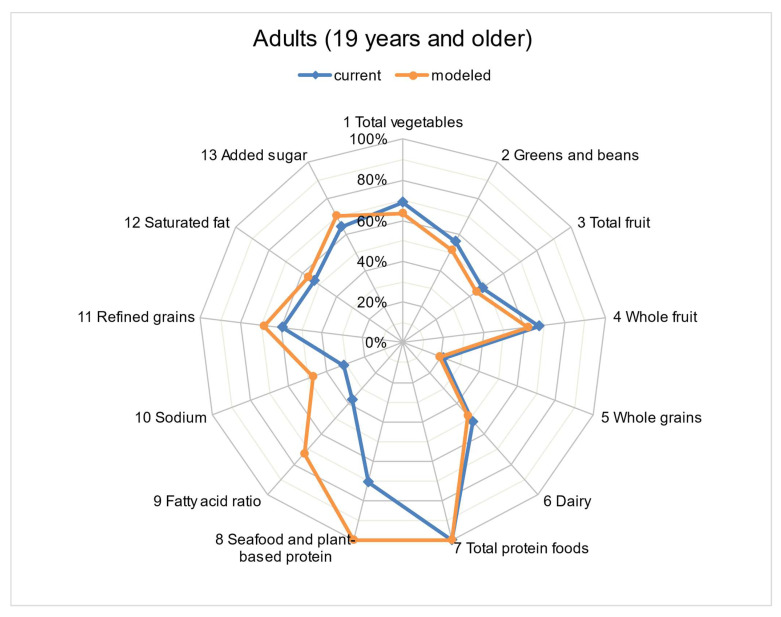
Radar plot to compare HEI-2015 * component scores (% of maximum possible score) for current intake and modeled intake (addition of 1 ounce of walnuts) among adults aged >19 years. * HEI-2015 includes 13 dietary components. Nine adequacy components (those recommended for inclusion in a healthy diet) include total vegetables (5), greens and beans (5), total fruits (5), whole fruits (5), whole grains (10), dairy (10), total protein foods (5), seafood and plant proteins (5), and fatty acids ratio (10). Four moderation components (those that should be consumed sparingly) include sodium (10), refined grains (10), saturated fats (10), and added sugars (10) (21).

The mean HEI-2015 scores of the usual (current) diet decreased with increasing age among children but improved with increasing age among adults (Table 5). In our model, the addition of 1 ounce (28.35 g) of walnuts to the diet significantly improved the diet quality for all age and sex groups. The diet quality assessed for the usual (current) intake did not achieve full scores for any of the 13 HEI-2015 components among children aged 4–18 years (Figure 2). However, the model that added 1 ounce (28.35 g) of walnuts to the usual (current) dietary intake obtained maximum scores for the categories “Total Protein Foods” and “Seafood and Plant Proteins”, while significantly improving the scores for “Fatty Acids” from 2.8 (95% CI: 2.5–3.0) to 6.5 (95% CI: 6.2–6.8) out of a total of 10 points (Table 2) among children aged 4–18 years. Among adults aged > 19 years, diet quality assessments for the usual (current) intake did not attain full scores in any of the HEI-2015 components except “Total Protein Foods” (Figure 3).” However, in our model, adding 1 ounce (28.35 g) of walnuts to the usual (current) dietary intake resulted in maximum scores for “Seafood and Plant Proteins”, and significantly improved the scores for “Fatty Acids” from 3.8 (95% CI: 3.5–4.0) to 7.3 (95% CI: 7.0–7.6) out of a total of 10 points among adults aged > 19 years.

## 4. Discussion

In the current study, walnut consumers were more likely to be older in age, female, white, and better educated, with a higher income, which is consistent with the findings of another study [28]. The HEI-2015 score, a measure of diet quality and adherence to the Dietary Guidelines for Americans recommendations [21,22], is based on nine encouraged food components and four components recommended for moderation. Improved diet quality has been linked to reductions in various chronic diseases and associated mortality risks [29,30,31,32,33]. In the current modeling study, the addition of 1 ounce (28.35 g) of walnuts to the regular diets of no-nut consumers from the NHANES significantly improved the diet quality among children, adolescents, and adults, as assessed by the HEI-2015. Furthermore, the inclusion of 1 ounce (28.35 g) of walnuts in the diet led to improvements in multiple dietary components of the HEI, which agree with previous observational studies that have reported associations between the consumption of nuts and higher diet quality in adults and children [34,35,36]. In this modeling study, significant increases were identified for two individual HEI components (seafood and plant proteins and the ratio of unsaturated to saturated fatty acids) for all age and sex groups.

One concern that has been raised when adding 1 ounce (28.35 g) of walnuts to the typical diet is the significant increase in caloric intake. Other studies have reported that walnut consumption improves health outcomes without changing body mass index [29,37], suggesting that the benefits of adding walnuts to the typical diet outweigh the concerns associated with a higher caloric intake. The protein and fiber contents of nuts may contribute to satiety and may serve as a mechanism for improving children’s diet quality by reducing their consumption of less-nutritious calories [36]. Walnuts are also rich in ALA, monounsaturated fatty acids, and essential vitamins and nutrients [3,4], and are healthier than many alternative snack foods that are high in saturated fatty acids, sugar, and salt.

In this modeling study, adding 1 ounce (28.35 g) of walnuts to the diet increased magnesium consumption by 16.9%, partially offsetting the low magnesium intakes among U.S. adults (aged > 19 years), and increased magnesium intake among children aged 4–18 years by 20.4% (Table 2). However, even after the addition of 1 ounce (28.35 g) of walnuts to the diet, up to 52% of adults and 34.6% of children and adolescents failed to achieve an adequate magnesium intake (Table 3). Ensuring an adequate magnesium intake is important for bone remodeling [38] and energy metabolism [39]; therefore, other magnesium-rich foods, such as vegetables and beans, should also be considered to help close this nutrient gap [40].

A significant portion of adults, especially adult women, do not currently achieve the recommended intake of folate. Folate is a key nutrient and has been added to grains in the U.S. to prevent congenital disabilities, especially neural tube defects [41]. In this modeling study, adding 1 ounce (28.35 g) of walnuts to the diet improved folate adequacy, but 17.2% of children aged 4–18 years and 32.2% of adults >19 years continued to present with inadequate levels of folate intake. These low levels of folate can be overcome by increasing the intake of folate-rich dark green leafy vegetables [40].

Although deficiencies in micronutrients, such as copper and zinc, are not considered widespread concerns in the U.S., adding 1 ounce (28.35 g) of walnuts to the diet was able to completely remove copper inadequacy and significantly improve zinc inadequacy for most of the populations in our model. Copper deficiency can result in blood vessel defects, iron-deficient anemia, osteoporosis and joint problems, brain disturbances, loss of skin or hair pigment, weakness, fatigue, skin sores, and poor thyroid function [42]. Zinc plays critical roles in immune function, cell division, protein and DNA synthesis, wound healing, normal growth, and development throughout lifecycle stages [43]. Having adequate levels of both copper and zinc are essential to health and including small amounts of walnuts in the diet could serve as an effective tool to increase the dietary intake of these micronutrients.

Walnuts are a rich source of ALA, the plant-based omega-3 fatty acid [44]. ALA has anti-inflammatory and other potentially beneficial properties that contribute to improving vascular endothelial function [45]. In one study, walnut consumption was more strongly related to a lower risk of cardiovascular disease than total nut consumption and was associated with a lower risk of stroke [46].

Vitamin E deficiency is a widespread concern among the U.S. population, varying from 56.8 to 95.2% (Table 3). In this modeling study, adding 1 ounce (28.35 g) of walnuts to the diet did not significantly improve vitamin E inadequacy. The term ‘vitamin E’ comprises four tocopherols (alpha-tocopherol, beta-tocopherol, gamma-tocopherol, and delta-tocopherol), but only alpha-tocopherol meets the criteria to be considered a vitamin. With a healthy gastrointestinal tract, tocopherols are available as small fatty molecules that are absorbed along with dietary fat in the intestine and enter the circulation via chylomicron particles [47]. Walnuts are an excellent source of gamma-tocopherol, supplying 21 mg/100 g [48]. Additionally, the hydroxylation and oxidation of gamma-tocopherol but not alpha-tocopherol in the liver generate potent free radical scavengers able to reduce pro-inflammatory eicosanoids and subsequent inflammatory responses. Therefore, gamma-tocopherol, not alpha-tocopherol, has been suggested to act as the cardioprotective form of vitamin E [47]. Similar benefits for gamma-tocopherol rather than alpha-tocopherol have also been observed in experimental and epidemiological cancer studies [49]. Therefore, although adding walnuts to the diet may not satisfy the dietary requirement for vitamin E, walnuts may confer health benefits due to high gamma-tocopherol contents, contributing to the resolution of inflammation.

In the current modeling study, adding walnuts to the usual diet significantly improved fiber intake across all age and gender categories, while significantly improving potassium intake only for people aged 4–18 years. However, adding 1 ounce (28.35 g) of walnuts to the diet had minimal effects on meeting the recommendation for fiber and potassium intake. Although walnuts contribute more of these two nutrients than other energy-dense snacks [35,50], the data from this study indicate that increasing a single food group is not sufficient to achieve the recommended levels of fiber and potassium intake based on current diet patterns. 

While adding a small amount of walnuts to the diet (i.e., the 1 ounce (28.35 g) in this modeling study) may improve the intake of some specific nutrients and diet quality, additional dietary strategies remain necessary to encourage healthy dietary intake and improve overall health. 

A major limitation of this study is that self-reported 24 h dietary recall data in the NHANES are subject to measurement errors due to large day-to-day variations in food intake. However, the NCI method was applied to reduce the measurement error and improve usual intake estimates. In this study, walnuts were added to the regular diet of no-nut consumers. If walnuts are consumed instead of high energy-dense, low nutrient-dense snacks, this could have large impacts on the overall dietary profile that are unable to be addressed in the current model. 

Another limitation of this study is the lack of individual-level data to obtain estimates for performing nominal tests of significance. In this study, the use of non-overlapping confidence intervals in comparing group means were permissible. However, these tests based on non-overlapping confidence intervals have been shown to be too conservative [51,52]. 

The strengths of this study include the use of a large, nationally representative database to examine food and nutrient intakes, and the application of advanced statistical techniques to assess the usual intake across numerous groups stratified by age and sex. 

## 5. Conclusions

Food-based recommendations aim to promote overall health and well-being by achieving optimal nutrient and energy intake levels. In this modeling study, adding 1 ounce (28.35 g) of walnuts to the usual dietary intake of no-nut consumers improved the diet quality among children, adolescents, and adults, in addition to improving the intake of some under-consumed nutrients. While walnut consumption is already promoted in dietary guidelines, the current study demonstrates the benefits of adding only 1 ounce of walnuts to the U.S. diet, which is a simple change that consumers could likely make. Further, the change in dietary nutrient profiles in response to adding this small amount of walnuts to the diet provides some insights into potential changes that may occur in the health profiles of individuals. This information can be useful for dietitians and health professionals to further emphasize the benefit of small dietary modulations that can be beneficial to consumers when promoting healthy dietary habits that include walnuts and can benefit overall health.

## Figures and Tables

**Table 1:** Weighted demographic characteristics of National Health and Nutrition Examination Survey (NHANES, 2015–2016 and 2017–2018 cycles) participants according to walnut, other nut, and no-nut consumption.

	Overall (*n* = 13,877)		Walnut Consumers (*n* = 933)	Other Nut Consumers (*n* = 5187)	No-Nut Consumers (*n* = 7757)	*p* Value ^c^
	N (Unweighted)	N (in 1000) (Weighted)	% (Weighted)	% (Weighted)	% (Weighted)	
**Age group**						<0.0001
4 to 8 years	1439	38,903	4.5%	41.6%	53.9%	
9 to 13 years	1479	41,157	6.8%	35.7%	57.5%	
14 to 18 years	1374	41,637	5.7%	33.4%	60.9%	
19 to 50 years	4717	247,556	7.6%	41.9%	50.5%	
51 to 70 years	3376	156,082	11.6%	47.6%	40.8%	
71 years and older	1492	58,701	14.5%	46.9%	38.6%	
**Sex**						<0.0001
Men	6832	287,249	7.8%	40.8%	51.4%	
Women	7045	296,787	10.1%	44.9%	45.0%	
**Race/Hispanic origin**						<0.0001
Mexican American and Other Hispanic	3848	100,466	5.5%	32.5%	62.0%	
Non-Hispanic White	4644	355,400	11.0%	47.8%	41.2%	
Non-Hispanic Black	3089	67,538	3.8%	32.6%	63.7%	
Other Race	2296	60,633	8.3%	43.0%	48.7%	
**Annual household income**						<0.0001
less than $20,000	2470	72,634	4.1%	31.3%	64.6%	
$20,000 to $75,000	6755	258,406	8.3%	41.3%	50.4%	
$75,000 to $9999	1323	69,711	10.6%	47.4%	42.0%	
over $100,000	2428	153,838	12.3%	50.5%	37.2%	
**Ratio of family income to poverty:** mean ± SD or SE	2.38 ± 1.59 ^a^	2.92 ± 0.06 ^b^	3.52 ± 0.10 ^b^	3.23 ± 0.06 ^b^	2.52 ± 0.06 ^b^	<0.0001
**Education**						<0.0001
Less than 9th grade	3665	96,643	5.6%	35.7%	58.8%	
9–11th grade (Incl. 12th grade with no diploma)	1894	60,535	4.7%	33.4%	61.9%	
High school graduate/GED or equivalent	2406	119,513	8.3%	36.7%	54.9%	
Some college or associates degree	3029	149,525	9.6%	43.6%	46.8%	
College degree or above	2322	142,650	13.4%	56.6%	30.0%	

^a^ Standard deviation = SD. ^b^ Standard error = SE. ^c^ Rao-Scott Chi2 test for categorical variables, linear regression for continuous variables.

**Table 2:** Under-consumed nutrients, protein intake, and energy intake for usual (current) and modeled dietary intakes (addition of 1 ounce of walnuts, mean (95% CI).

Nutrients	4–18 Years (*n* = 2670)	19–50 Years (*n* = 2669)	51–70 Years (*n* = 1711)	>71 Years (*n* = 707)
Magnesium, mg/day				
Current	219.2 (214.2–224.2)	270.4 (262.1–278.8)	264.1 (255.5–272.6)	232.4 (224.3–240.6)
Modeled	263.9 (259.0–268.9) *	315.0 (306.7–323.3) *	308.5 (300.1–317.0) *	277.5 (269.3–285.7) *
Calcium, mg/day				
Current	941.4 (912.1–970.8)	929.8 (897.2–962.5)	858.5 (816.7–900.3)	760.8 (714.4–807.2)
Modeled	969.3 (940.0–998.6)	957.1 (924.5–989.8)	886.1 (844.4–927.8)	788.9 (742.6–835.3)
Potassium, mg/day				
Current	2070 (2019–2121)	2438 (2365–2510)	2509 (2430–2588)	2289 (2212–2366)
Modeled	2195 (2144–2246)*	2561 (2489–2633)	2633 (2554–2711)	2414 (2337–2491)
Folate, µg DFE/d				
Current	347.5 (338.2–356.8)	369.0 (356.2–381.8)	346.2 (334.3–358.1)	319.2 (302.5–336.0)
Modeled	375.3 (366.0–384.5) *	396.6 (383.9–409.3) *	373.4 (361.6–385.2) *	346.9 (330.0–363.7)
Vitamin E, mg/day				
Current	6.6 (6.4–6.8)	7.7 (7.5–8.0)	7.2 (6.9–7.5)	6.6 (6.3–6.9)
Modeled	6.8 (6.6–7.0)	7.9 (7.7–8.2)	7.4 (7.1–7.7)	6.8 (6.5–7.1)
Fiber, g/day				
Current	13.6 (13.2–13.9)	14.7 (14.0–15.4)	14.6 (14.0–15.2)	13.8 (13.1–14.5)
Modeled	15.4 (15.1–15.8) *	16.6 (15.9–17.3) *	16.5 (15.9–17.1) *	15.7 (15.0–16.4) *
Omega-3 fatty acids, g/day				
Current	1.41 (1.37–1.46)	1.76 (1.70–1.82)	1.66 (1.58–1.73)	1.48 (1.38–1.58)
Modeled	3.98 (3.93–4.03) *	4.33 (4.27–4.38) *	4.23 (4.16–4.30) *	4.08 (3.97–4.19) *
Protein, g/day				
Current	67.2 (65.4–68.9)	82.3 (79.8–84.9)	77.2 (74.4–80.1)	63.9 (61.3–66.5)
Modeled	71.5 (69.7–73.2) *	86.6 (84.0–89.2)	81.6 (78.8–84.4)	68.2 (65.7–70.8)
Energy, kcal/day				
Current	1856 (1817–1896)	2157 (2111–2203)	1989 (1915–2062)	1651 (1593–1710)
Modeled	2041 (2002–2081) *	2339 (2294–2385) *	2172 (2100–2245) *	1838 (1780–1896) *

* The 95% confidence intervals of mean values for current intake and modeled intake do not overlap.

**Table 3:** Percentage of individuals with nutrient intakes below the EAR ^a^ at usual (current) and modeled intakes (addition of 1 ounce of walnuts).

	Calcium		Magnesium		Folate		Vitamin E	
Age Group	Usual Diet % (95% CI)	After Modeling % (95% CI)	Usual Diet % (95% CI)	After Modeling % (95% CI)	Usual Diet % (95% CI)	After Modeling % (95% CI)	Usual Diet % (95% CI)	After Modeling % (95% CI)
**Boys**								
4–8 years (*n* = 424)	33.7 (27.5–39.9)	29.9 (23.9–35.9)	4.4 (2.9–5.9)	0.3 (0.1–0.5) *	4.0 (1.5–6.5)	1.6 (0.2–2.9)	56.8 (51.0–62.6)	53.5 (47.7–59.3)
9–13 years (*n* = 452)	66.8 (62.0–71.6)	64.2 (59.2–69.1)	41.2 (35.2–47.1)	18.3 (13.7–23.0) *	19.1 (14.7–23.6)	12.4 (8.7–16.2)	77.9 (74.5–81.3)	76.5 (73.0–80.0)
14–18 years (*n* = 456)	64.8 (60.6–69.0)	62.4 (57.9–66.9)	82.7 (79.6–85.9)	71.6 (67.6–75.6) *	44.6 (38.8–50.4)	37.1 (31.3–43.0)	88.2 (85.2–91.1)	87.5 (84.5–90.6)
Total (Boys) (*n* = 1332)	56.0 (52.9–59.2)	53.1 (49.9–56.4)	45.7 (43.3–48.0)	32.9 (30.7–35.1) *	24.2 (20.7–27.6)	18.4 (15.2–21.7)	75.4 (72.7–78.1)	73.7 (70.9–76.5)
**Girls**								
4–8 years (*n* = 422)	42.3 (36.9–47.8)	38.1 (32.9–43.4)	6.3 (4.7–7.8)	0.4 (0.2–0.7) *	5.6 (3.0–8.1)	2.3 (1.0–3.6)	59.9 (56.1–63.8)	56.5 (52.9–60.1)
9–13 years (*n* = 485)	75.3 (70.7–79.9)	72.8 (68.1–77.5)	50.6 (45.2–55.9)	24.3 (19.7–28.9) *	24.5 (19.9–29.1)	16.0 (12.3–19.7) *	82 (78.7–85.2)	80.6 (77.2–83.9)
14–18 years (*n* = 431)	82.1 (77.6–86.6)	80.4 (75.8–85.0)	89.0 (85.9–92.0)	78.3 (74.6–82.1) *	63.9 (59.1–68.7)	55.2 (50.0–60.3)	95.2 (92.7–97.6)	94.8 (92.2–97.4)
Total (Girls) (*n* = 1338)	68.1 (64.8–71.4)	65.4 (62.1–68.6)	51.2 (48.3–54.1)	36.4 (33.5–39.3) *	32.9 (29.5–36.3)	25.8 (22.8–28.9) *	80.2 (78.0–82.4)	78.6 (76.4–80.8)
Total (Boys and Girls) (*n* = 2670)	61.8 (59.2–64.4)	59.0 (56.3–61.6)	48.3 (46.1–50.5)	34.6 (32.7–36.5) *	28.3 (25.7–30.9)	22.0 (19.5–24.4) *	77.7 (75.7–79.7)	76.0 (74.0–78.1)
**Adults**								
19–50 years								
Men (*n* = 1367)	35.1 (31.5–38.6)	32.1 (28.7–35.5)	66.0 (62.7–69.2)	51.1 (47.4–54.7) *	35.6 (31.7–39.5)	28.3 (24.7–31.8)	81.5 (78.8–84.2)	80.7 (77.9–83.5)
Women (*n* = 1302)	57.1 (53.0–61.2)	53.6 (49.3–57.9)	65.2 (61.5–69.0)	43.4 (39.7–47.1) *	58.4 (54.3–62.6)	49.2 (45.2–53.2) *	93.0 (91.5–94.5)	92.5 (91.0–94.1)
Total (*n* = 2669)	45.1 (41.9–48.3)	41.9 (38.7–45.1)	65.6 (62.7–68.6)	47.6 (44.5–50.7) *	46.0 (42.8–49.2)	37.8 (34.7–40.8) *	86.7 (84.8–88.6)	86.1 (84.1–88.0)
51–70 years								
Men (*n* = 896)	41.0 (36.0–46.0)	37.6 (32.8–42.4)	73.0 (69.5–76.4)	59.9 (56.2–63.6) *	39.9 (36.6–43.2)	31.8 (28.7–34.8) *	84.8 (82.2–87.5)	84.0 (81.3–86.8)
Women (*n* = 815)	82.7 (78.5–87.0)	80.9 (76.5–85.3)	72.3 (67.7–76.9)	51.5 (46.3–56.7) *	65.3 (61.2–69.4)	56.2 (51.6–60.7) *	95.1 (92.2–98.1)	94.8 (91.8–97.8)
Total (*n* = 1711)	61.2 (57.2–65.3)	58.6 (54.5–62.7)	72.6 (70.1–75.2)	55.8 (52.7–59.0) *	52.2 (49.5–54.9)	43.6 (40.6–46.6) *	89.8 (87.8–91.8)	89.2 (87.2–91.2)
>71 years (*n* = 707)	81.4 (77.3–85.5)	79.4 (74.8–84.0)	82.8 (80.2–85.5)	65.9 (61.9–69.9) *	58.2 (52.3–64.2)	48.1 (41.9–54.4)	94.7 (93.3–96.1)	94.3 (92.9–95.8)
Total (Men) (*n* = 2634)	40.3 (37.4–43.1)	37.3 (34.5–40.1)	69.8 (67.5–72.2)	55.9 (53.2–58.6) *	38.0 (35.2–40.8)	30.2 (27.5–32.9) *	83.3 (81.4–85.2)	82.6 (80.6–84.5)
Total (Women) (*n* = 2453)	68.9 (65.7–72.2)	66.1 (62.7–69.6)	69.3 (66.5–72.1)	47.8 (44.8–50.7) *	61.6 (58.5–64.7)	52.2 (49.1–55.4) *	94.2 (92.8–95.7)	93.8 (92.4–95.3)
Total (All Adults) (*n* = 5087)	53.8 (51.3–56.3)	50.9 (48.4–53.4)	69.6 (67.4–71.8)	52.0 (49.6–54.4) *	49.2 (47.1–51.3)	40.6 (38.5–42.8) *	88.5 (87.2–89.8)	87.9 (86.5–89.3)

* The 95% confidence intervals of values of those variables below recommended levels for current intake and modeled intake do not overlap. ^a^ EAR, estimated average requirement: a nutrient intake value that is estimated to meet the requirement of half the healthy individuals in a group [26].

**Table 4:** Percentage of individuals with nutrient intakes above the AL ^a^ at usual (current) and modeled intakes (addition of 1 ounce of walnuts).

	Potassium		Fiber	
Age Group	Usual Diet % (95% CI)	After Modeling % (95% CI)	Usual Diet % (95% CI)	After Modeling % (95% CI)
**Boys**				
4–8 years (*n* = 424)	31.7 (26.4–37.0)	37.8 (32.2–43.3)	1 (0.4–1.7)	0.6 (0.1–1.1)
9–13 years (*n* = 452)	29.5 (24.9–34.0)	34.6 (29.5–39.7)	0.8 (0.3–1.3)	0.5 (0.1–0.9)
14–18 years (*n* = 456)	20.4 (15.8–25.1)	23.6 (18.7–28.5)	0.4 (0.1–0.8)	0.2 (0.0–0.5)
Total (Boys) (*n* = 1332)	26.7 (24.0–29.5)	31.4 (28.5–34.3)	0.7 (0.2–1.2)	0.4 (0.1–0.8)
**Girls**				
4–8 years (*n* = 422)	21.8 (18.1–25.4)	27.5 (23.5–31.5)	2 (1.0–3.0)	1.2 (0.4–1.9)
9–13 years (*n* = 485)	27.8 (23.4–32.2)	33.8 (28.9–38.8)	1.1 (0.5–1.8)	0.7 (0.3–1.1)
14–18 years (*n* = 431)	21.8 (17.1–26.4)	26.5 (21.5–31.5)	0.9 (0.3–1.4)	0.5 (0.1–0.9)
Total (Girls) (*n* = 1338)	23.9 (21.5–26.4)	29.4 (26.7–32.0) *	1.3 (0.6–2.0)	0.8 (0.3–1.2)
Total (Boys and Girls) (*n* = 2670)	25.4 (23.3–27.5)	30.4 (28.2–32.7) *	1 (0.4–1.6)	0.6 (0.2–1.0)
**Adults**				
19–50 years				
Men (*n* = 1367)	22.9 (20.3–25.6)	25.8 (22.9–28.7)	0.4 (0.1–0.8)	0.2 (0.0–0.5)
Women (*n* = 1302)	22.8 (19.6–26.1)	27.1 (23.7–30.5)	0.7 (0.2–1.2)	0.5 (0.1–0.8)
Total (*n* = 2669)	22.9 (20.6–25.2)	26.4 (23.9–28.9)	0.6 (0.1–1.0)	0.3 (0.1–0.6)
51–70 years				
Men (*n* = 896)	25.3 (21.8–28.8)	28.2 (24.7–31.7)	0.6 (0.1–1.2)	0.4 (0.0–0.7)
Women (*n* = 815)	23.9 (19.5–28.2)	28.6 (23.9–33.3)	1.4 (0.6–2.1)	0.8 (0.3–1.4)
Total (*n* = 1711)	24.6 (22.3–26.9)	28.4 (25.9–30.9)	1 (0.3–1.6)	0.6 (0.2–1.0)
>71 years (*n* = 707)	17.3 (14.5–20.1)	21.3 (18.2–24.4)	2.4 (1.1–3.7)	1.7 (0.7–2.6)
Total (Men) (*n* = 2634)	22.9 (20.9–25.0)	25.8 (23.6–28.0)	0.6 (0.2–1.0)	0.4 (0.0–0.7)
Total (Women) (*n* = 2453)	22.7 (20.5–24.9)	27.2 (24.9–29.6)	1.2 (0.6–1.8)	0.8 (0.4–1.2)
Total (All Adults) (*n* = 5087)	22.8 (21.1–24.5)	26.5 (24.6–28.4) *	0.9 (0.4–1.4)	0.6 (0.2–0.9)

* The 95% confidence intervals of values from those variables above the adequate intake (AI) for usual (current) intake and modeled intake do not overlap. ^a^ AI is a recommended average daily nutrient intake level, based on experimentally derived intake levels or approximations of observed mean nutrient intake by a group (or groups) of apparently healthy people that are assumed to be adequate [26].

**Table 5:** Diet quality scores (HEI scores) for usual (current) and modeled intakes (addition of 1 ounce of walnuts).

	HEI Score ^a^ (Mean (95% CI))	
Age Group	Usual Diet	After Modeling
Boys		
4–8 years	51.4 (48.93–53.77)	61.4 (59.17–63.73) *
9–13 years	46.8 (44.26–49.34)	56.4 (54.09–58.76) *
14–18 years	44.6 (42.42–46.79)	53.3 (51.31–55.22) *
Total (Boys)	47.3 (45.67–48.94)	56.5 (55.00–58.12) *
Girls		
4–8 years	54.1 (52.23–55.97)	65.2 (63.27–67.08) *
9–13 years	51.6 (49.10–54.19)	60.8 (58.59–63.03) *
14–18 years	48.7 (45.88–51.46)	58 (55.62–60.41) *
Total (Girls)	51.5 (50.06–53.07)	61.1 (59.88–62.33) *
Total (Boys and Girls)	49.1 (47.94–50.37)	58.5 (57.52–59.56) *
Adults		
19–50 years		
Men	49.7 (47.86–51.65)	56.2 (54.69–57.78) *
Women	50.7 (48.45–53.02)	58.4 (56.40–60.48) *
Total	50.1 (48.36–51.87)	57.1 (55.68–58.54) *
51–70 years		
Men	54 (51.09–56.98)	59.7 (57.11–62.42) *
Women	56.4 (54.66–58.14)	63.7 (62.04–65.42) *
Total	55 (53.00–56.93)	61.4 (59.52–63.32) *
>71 years	59.6 (56.37–62.67)	66.5 (63.78–69.14) *
Total (Men)	51.7 (50.14–53.18)	57.9 (56.57–59.25) *
Total (Women)	53.5 (51.86–55.16)	61.1 (59.50–62.69) *
Total (All Adults)	52.4 (50.99–53.79)	59.2 (57.95–60.50) *

* The 95% confidence intervals of mean HEI scores for current intake and modeled intake do not overlap. ^a^ The Healthy Eating Index (HEI) is a diet quality index (scores ranging from 0 to 100) that measures alignment of diet with the Dietary Guidelines for Americans.

## Data Availability

Data used in the manuscript are available at https://www.cdc.gov/nchs/nhanes/index.htm. accessed on 10 November 2021. The analytic code will be made available upon request.
